# Transcriptomic Responses of *Sclerodermus alternatusi* Yang to Ultraviolet (UV) Stress of Different Wavelengths

**DOI:** 10.3390/ijms27031163

**Published:** 2026-01-23

**Authors:** Fei Li, Wenting Jin, Huan Cheng, Fengyuan Wu, Yufei Pan, Denghui Zhu, Shan Xu, Cao Zhou, Bingchuan Zhang, Amrita Chakraborty, Amit Roy, Shulin He

**Affiliations:** 1 College of Life Sciences, Chongqing Normal University, Shapingba District, Chongqing 401331, China; leef0925@cqnu.edu.cn (F.L.); xushancqnu@126.com (S.X.); zhouc@cqnu.edu.cn (C.Z.); zhangbingchuan@cqnu.edu.cn (B.Z.); 2 Chongqing—Rwanda Belt and Road Joint Laboratory for Integrated Pest Management of Fruits Vegetables and Grains, Chongqing Normal University, Shapingba District, Chongqing 401331, China; 3 Chongqing Key Laboratory of Vector Insects, College of Life Sciences, Chongqing Normal University, Shapingba District, Chongqing 401331, China; 4 Faculty of Forestry and Wood Sciences, Czech University of Life Sciences Prague, 129 Kamýcká, 165 00 Prague, Czech Republic; chakraborty@fld.czu.cz

**Keywords:** ultraviolet stress, *Sclerodermus alternatusi* Yang, RNA-Seq, RT-qPCR

## Abstract

Ultraviolet (UV) radiation is a significant environmental stressor that exerts profound impacts on insect physiology, behaviour and survival. Although some insects can use UV light for spatial orientation and navigation, it can induce DNA damage, oxidative stress, and impair critical biological functions, ultimately reducing ecological fitness. *Sclerodermus alternatusi* Yang (Hymenoptera: Bethylidae) is a dominant ectoparasitoid of the early instar larvae of *Monochamus alternatus* and plays a key role in the biological control of this pest in forestry systems; however, it faces intense UV exposure in the field environment. Despite its ecological importance, the molecular mechanisms underlying its responses to UV-induced stress remain poorly understood. In this study, newly emerged adult wasps (within 24 h post-eclosion) were exposed to UVA (365 nm) and UVC (253.7 nm) radiation for 9 h under controlled laboratory conditions. Total RNA was extracted from treated and control individuals for transcriptomic analysis using RNA-Seq. A total of 505 differentially expressed genes (DEGs) were identified; gene ontology enrichment analysis revealed that UVA exposure significantly upregulated genes involved in cellular respiration and oxidative phosphorylation, suggesting an enhanced metabolic response. Furthermore, Kyoto Encyclopedia of Genes and Genomes (KEGG) pathway enrichment analysis revealed that UV stress modulates energy metabolism through the activation of oxidative phosphorylation and thermogenesis-related pathways, highlighting the reallocation of energy resources in response to UV-induced stress. To validate the RNA-Seq data, four representative DEGs were selected for quantitative real-time PCR (RT-qPCR) analysis. The qPCR results were consistent with the transcriptomic trends, confirming the reliability of the sequencing data. Collectively, this study provides a comprehensive overview of the molecular response mechanisms of *S. alternatusi* to UV stress, offering novel insights into its environmental adaptability and laying a theoretical foundation for its application in biological pest control under field conditions.

## 1. Introduction

Ultraviolet (UV) radiation is a significant environmental stressor that can be categorised into three types based on wavelength: UVA (320–400 nm), UVB (280–320 nm), and UVC (100–280 nm). Insects are routinely exposed to UV radiation in natural environments and have evolved physiological and behavioural adaptations to cope with this stress. Previous studies have shown that UV exposure can interfere with insect stress detoxification, longevity, and reproduction, as well as gene expression and immune responses [1,2,3]. Additionally, certain insects utilise the UV portion of the solar spectrum for spatial orientation. Among the UVs, UVC and most UVB are effectively absorbed by atmospheric oxygen and ozone, resulting in predominantly UVA radiation at ground level [4]. However, UVC is frequently employed in laboratory studies as a high-intensity stressor to model DNA damage and cellular responses in insects [5,6]. Generally, long-wavelength UV radiation (UVA) primarily induces oxidative stress and protein denaturation. In contrast, short-wavelength UVC radiation is confined mainly to superficial tissue layers due to its limited penetration depth and is more likely to cause DNA damage, inhibit RNA polymerase II elongation, and initiate transcription-coupled DNA repair mechanisms [7,8]. For instance, UVA exposure has been reported to reduce adult longevity and offspring emergence rates in *Trichogramma* spp. [9]. Additionally, UVA can increase oxidative stress levels in various insect species, such as *Belgica antarctica*, *Helicoverpa armigera*, and *Tribolium castaneum* [10,11]. Notably, *Ips nitidus*, a conifer-feeding bark beetle adapted to high-altitude environments, shows significant expansion in DNA damage repair genes, suggesting a key role in coping with UV radiation and hypoxia [12]. Interestingly, parasitoid wasps are hypothesised to be more sensitive to UV radiation due to their thinner chitinous cuticle [13].

*Sclerodermus alternatusi* Yang is a predominant parasitoid of *Monochamus alternatus* larvae and plays a crucial role in biological control strategies against this major forest pest [14,15]. This species exhibits sub-social behaviour and pronounced aggressiveness, resulting in up to 95.66% mortality in *M. alternatus* larvae at instars I–III, thereby demonstrating significant advantages over other parasitoid species [16]. Large-scale artificial rearing of *S. alternatusi* has been successfully achieved, typically using *Thyestilla gebleri* as an alternative host [17,18], highlighting its practical potential in forest pest management programs. Despite its promising biocontrol application, molecular studies on *S. alternatusi* remain limited. To date, only a few studies have been published. For example, Zhou et al. analysed the antennal transcriptome of *Sclerodermus* spp. and provided preliminary insights into olfactory-related gene families. However, systematic studies addressing the molecular responses of *S. alternatusi* to environmental stressors, such as UV radiation, are still lacking [19]. Recently, the availability of a chromosome-level genome assembly for *S. alternatusi* has opened new opportunities for investigating its molecular mechanisms of stress adaptation, providing a new path for downstream gene expression studies, and helping to improve its biocontrol efficacy and environmental resilience [20].

Although transcriptomic studies on insect responses to environmental stress are becoming increasingly prevalent, investigations focusing on *S. alternatusi*, particularly under UV stress, remain scarce. To address this knowledge gap, the present study employed RNA-Seq technology to characterise transcriptomic changes in *S. alternatusi* following exposure to UV radiation of different wavelengths. We further identified key pathways associated with energy metabolism and validated the expression of four representative differentially expressed genes (DEGs) using RT-qPCR. Our findings provide novel insights into the molecular mechanisms of UV stress adaptation in *S. alternatusi*, offering a theoretical foundation for improving its environmental fitness. While the UV intensity used in our laboratory study is higher than in natural environments, these results demonstrate adaptation of the parasitoid to varying UV conditions in the field, supporting its potential application in forest pest biocontrol.

## 2. Results

### 2.1. Quality Assessment of Transcriptome Sequencing Data Under Different UV Treatments

High-throughput RNA sequencing was performed on adult *S. alternatusi* at the same developmental stage using the Illumina sequencing platform. Samples were collected from three treatment groups: UVA-exposed, UVC-exposed, and a blank control (CK). Raw sequencing data were subjected to quality control using FastQC (v0.12.1, Babraham Bioinformatics, Babraham Institute, Cambridge, UK) and Fastp (v0.23.2, Chen Li, College of Life Sciences, Peking University, Beijing, China) and high-quality clean reads were aligned to the reference genome using HISAT2 (v2.2.1, John Hopkins University, Baltimore, MD, USA).

Across all samples, the number of filtered clean reads ranged from 19,689,673 to 24,045,284, with total clean bases ranging from 2.95 to 3.59 Gbp. The GC content remained stable, ranging from 37% to 39%, indicating a consistent nucleotide composition across the libraries. Genome alignment showed that over 98% of the reads from each sample were successfully mapped to the reference genome, suggesting high-quality sequencing data and excellent mapping efficiency. The alignment was performed using default parameters. These results confirmed the suitability of the data for downstream differential expression analysis and functional annotation (Table 1).

### 2.2. Global Transcriptomic Structure Analysis

**Figure 1:**
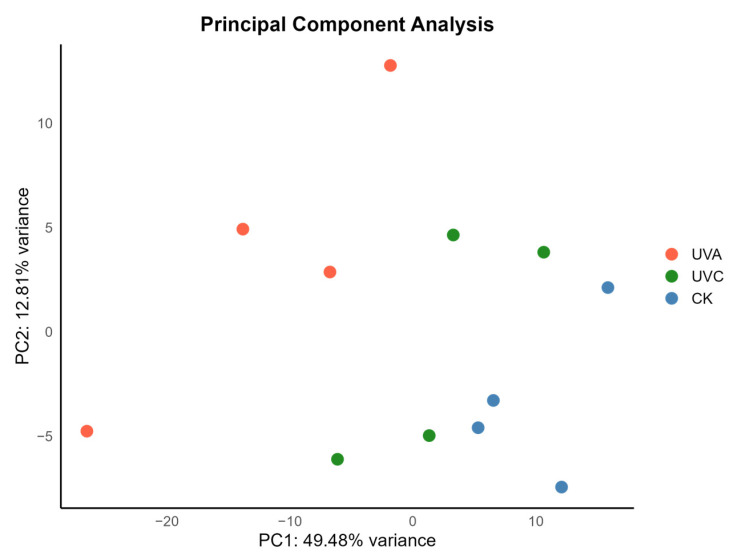
Principal Component Analysis (PCA) of the Transcriptome Profiles under UVA, UVC, and Control Treatments.

PCA revealed a clear separation among the three treatment groups along the first two principal components (PC1 and PC2), as shown in Figure 1. PC1 accounted for 49.48% of the total variance, while PC2 explained 12.81%. Specifically, samples from group A (UVA-treated) were distinctly separated from the CK along the PC1 axis, indicating substantial differences in gene expression profiles between these two groups. Group C (UVC-treated) exhibited moderate divergence from both group A and the control group, suggesting that UVC exposure also induced transcriptomic alterations. Biological replicates within each group were closely clustered, indicating high intra-group consistency and reproducibility. The overall data quality was robust, providing a solid foundation for subsequent differential expression and functional enrichment analyses.

### 2.3. Comparison of Differentially Expressed Genes in UV Treatment

**Figure 2:**
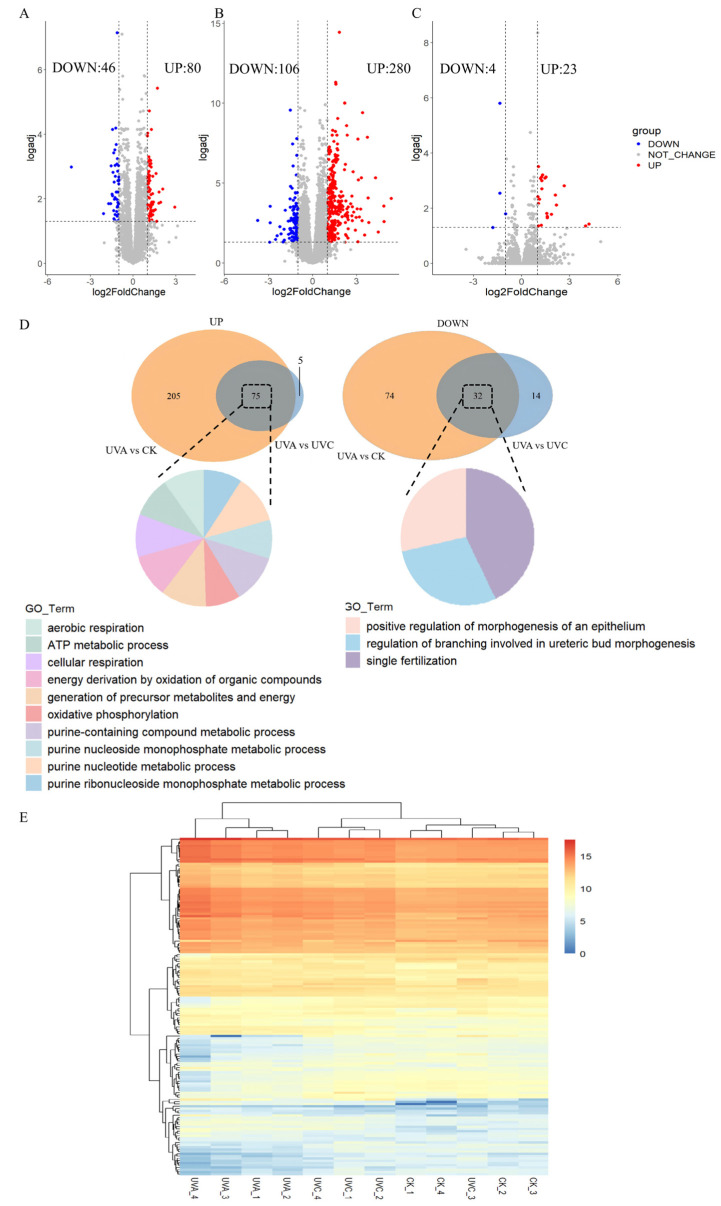
Volcano plots and Venn diagrams of DEGs in *S. alternatusi* under UVA and UVC treatments. (**A**) Volcano plot: UVA vs. UVC; (**B**) Volcano plot: UVA vs. CK; (**C**) Volcano plot: UVC vs. CK. In volcano plots, the vertical dotted lines represent the log_2_FoldChange, while the horizontal dotted lines indicate the threshold for logadj (log(adjusted *p*-value)); (**D**) Venn diagrams of common differential expression of upregulated (**left**) and downregulated (**right**) genes between UVA vs. CK and UVA vs. UVC comparisons. The pie charts indicate associated Gene Ontology (GO) terms. (**E**) Heatmap of DEGs showing upregulated and downregulated genes in UVA vs. UVC and UVC vs. CK comparisons.

To elucidate transcriptional alterations induced by different ultraviolet treatments, we performed differentially expressed genes (DEGs) analyses across three comparison groups: UVA vs. UVC, UVA vs. CK, and UVC vs. CK. In the UVA vs. UVC comparison, a total of 14,085 genes were detected, among which 46 genes were significantly downregulated, and 80 genes were significantly upregulated, indicating a relatively moderate transcriptomic divergence between these two conditions (Figure 2A). In contrast, the UVA vs. CK comparison revealed a substantially greater number of DEGs, including 106 downregulated and 280 upregulated genes, suggesting a more pronounced transcriptional response to UVA exposure relative to the untreated control (Figure 2B). Notably, the UVC vs. CK comparison yielded the fewest DEGs, with only 4 genes downregulated and 23 genes upregulated (Figure 2C). In addition, heatmaps were generated for the DEGs in UVA vs. UVC and UVC vs. CK, providing a clear visualisation of the expression pattern differences between treatments (Figure 2E).

Venn diagram-based intersection analyses between DEGs from the UVA vs. CK and UVA vs. UVC comparisons showed that 75 upregulated genes were shared between the two comparisons. In contrast, 205 upregulated genes were unique to UVA compared to CK, and 5 were specific to UVA compared to UVC. For downregulated genes, 32 genes were commonly downregulated in both comparisons. Additionally, 74 genes were uniquely downregulated in UVA compared to CK, and 14 were exclusive to UVA compared to UVC (Figure 2D).

### 2.4. GO and KEGG Enrichment Analyses of Differentially Expressed Genes

GO enrichment analysis of the DEGs between the UVA-treated and CK groups revealed that UVA exposure significantly altered the expression of genes associated with various biological functions. A total of 176 DEGs were enriched in GO terms, including 144 upregulated and 32 downregulated genes. These genes were distributed across three major GO categories: Biological Process (BP), Molecular Function (MF), and Cellular Component (CC). Among them, BP terms comprised the largest number of enriched entries, followed by MF and CC.

**Figure 3:**
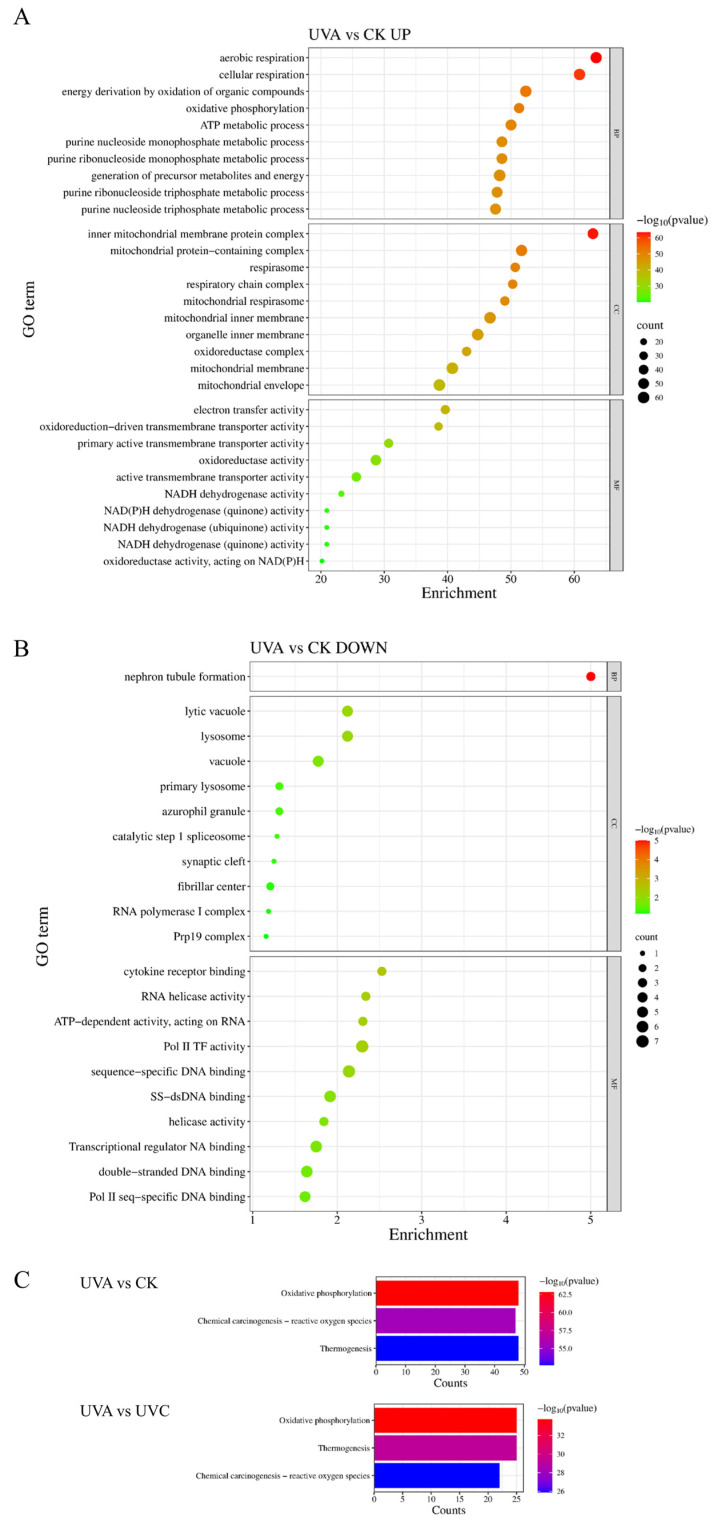
GO, and KEGG Enrichment Analysis of Differentially Expressed Genes (DEGs). (**A**) GO enrichment of upregulated genes in the UVA vs. CK comparison; (**B**) GO enrichment of downregulated genes in the UVA vs. CK comparison; (**C**) KEGG Enrichment Analysis of Upregulated Genes in UVA vs. CK and UVA vs. UVC Comparisons.

Among the upregulated DEGs, GO terms in the BP category were predominantly associated with mitochondrial energy metabolism, including aerobic respiration, cellular respiration, oxidative phosphorylation, and ATP synthesis coupled to electron transport. In the MF category, significantly enriched terms included electron transfer activity, oxidoreductase activity, and transmembrane transporter activity. In the CC category, the upregulated genes were primarily enriched in the mitochondrial inner membrane, respiratory chain complex, and mitochondrial protein-containing complex (Figure 3A).

Conversely, among the downregulated DEGs, enriched BP terms were mainly related to neuron tubule formation, suggesting suppression of developmental processes. In the MF category, downregulated genes were enriched in RNA helicase activity, transcription regulator activity, sequence-specific double-stranded DNA binding, and RNA polymerase II-specific DNA binding, reflecting decreased transcriptional regulation activity. A small subset of downregulated genes was also associated with lysosome-related components in the CC category (Figure 3B).

In the comparison between UVA and UVC treatments, GO enrichment results showed a consistent pattern: upregulated genes were significantly enriched in mitochondrial energy-related biological processes, including aerobic respiration and cellular respiration, and were localised to the mitochondrial membrane and respiratory chain complexes. In terms of MF, they were enriched in electron transfer activity and oxidoreductase-driven transmembrane transport. In contrast, downregulated genes in this comparison were primarily enriched in transcription factor activity, indicating a shift in gene regulatory networks.

To further elucidate pathway-level differences, KEGG pathway enrichment analysis was performed on upregulated DEGs in the UVA vs. CK and UVA vs. UVC comparisons. The results highlighted significant enrichment in several key pathways. Notably, oxidative phosphorylation and thermogenesis pathways were markedly upregulated in both comparisons, indicating enhanced mitochondrial bioenergetics under UVA stress (Figure 3C).

### 2.5. Validation of RNA-Seq Results by RT-qPCR

To further validate the accuracy and reliability of the RNA sequencing (RNA-seq) data, RT-qPCR was performed on a subset of representative DEGs identified in the UVA vs. CK comparison group. Four functionally significant genes involved in the ultraviolet stress response were selected for validation: *Alpha-ketoglutarate dehydrogenase complex 4* (*α-KGDHC*), *Glutathione S-transferase 1* (*GST 1*), *NADH dehydrogenase* (*ubiquinone*) *B22 subunit* (*ND-B22*), *Cytochrome P450 304a1* (*CYP450*). These genes are involved in critical biological processes, including redox homeostasis, antioxidant metabolism, energy transduction, and signal transduction pathways.

**Figure 4:**
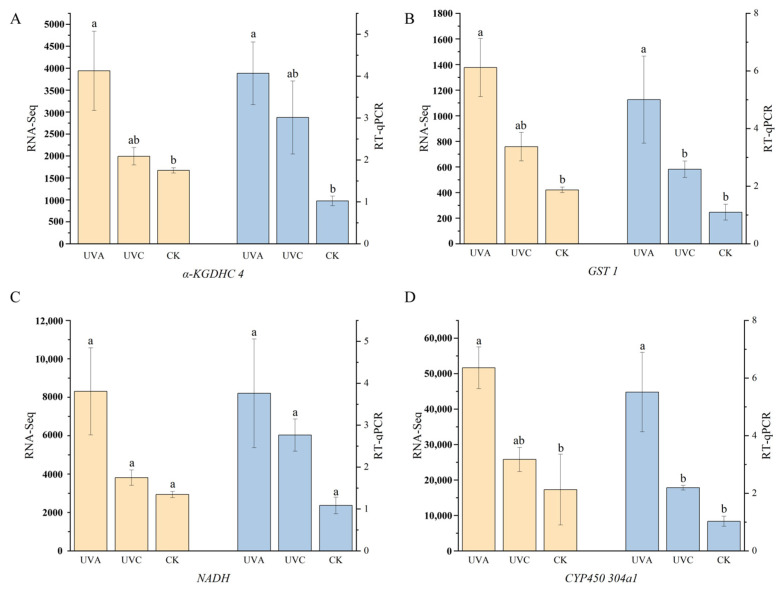
RT-qPCR Validation of DEGs Identified from RNA-Seq Analysis. (**A**) *α-KGDHC 4*; (**B**) *GST 1*; (**C**) *NADH*; (**D**) *CYP450 304a1*. For the same colour representing identical categories of data, the different height bars annotated with distinct letters indicate that, based on the one-way analysis of variance (ANOVA) followed by Scheffe’s post hoc test, significant differences exist between treatment groups (*p* < 0.05).

The RT-qPCR results indicate that, compared to the CK group, all four genes were significantly upregulated under UVA treatment, with varying degrees of increase observed under UVC exposure. The results of RT-qPCR and RNA-Seq analyses both demonstrated high concordance in the expression trends and magnitudes of change (Figure 4). Specifically, the expression levels of *GST 1*, *CYP450*, and *α-KGDHC* were significantly higher in the UVA group compared to the UVC and CK groups (*p* < 0.05). In contrast, no significant differences were observed for the *NADH* gene across the groups (*p* > 0.05). Overall, the expression patterns under both UVA and UVC stress conditions were highly consistent between RT-qPCR and RNA-Seq analyses, further validating the reliability and credibility of the transcriptomic data.

## 3. Discussion

Ultraviolet (UV) radiation, including both UVA and UVC, represents a significant environmental stressor that insects encounter [21]. A wealth of studies has explored the impact of UV radiation on various insect species. For instance, UV radiation has been shown to affect antioxidant enzyme activities in *T. castaneum* and alter metabolic and immune responses in *Drosophila melanogaster* [22]. However, despite considerable research on the effects of UV radiation on insect metabolism and development, the molecular response mechanisms in *S. alternatusi* under UV stress remain inadequately explored. In the present study, we systematically analysed the transcriptomic responses of *S. alternatusi* adults subjected to UVA and UVC irradiation. Our results revealed that UV stress significantly activated several pathways related to mitochondrial energy metabolism and electron transport, including aerobic respiration, oxidative phosphorylation, and ATP synthesis coupled to electron transport. These findings are consistent with the responses observed in other insect species exposed to UV radiation [23,24]. Notably, while UV exposure induced substantial metabolic reprogramming, no noticeable changes were observed in the external appearance of the insects under UV radiation exposure. Concurrently, pathways related to transcription factor activity and developmental processes, such as RNA polymerase II-associated transcription regulation and retinal/embryonic development, were significantly downregulated in response to UV exposure. These transcriptomic alterations suggest a regulatory paradigm centered on metabolic reprogramming and stress adaptation, potentially representing a widespread adaptive strategy employed by insects in response to UV stress.

Based on our experimental findings, we notably discovered that UVA exposure significantly upregulated key metabolic pathways, including oxidative phosphorylation, aerobic respiration, and mitochondrial electron transport, indicating a heightened state of mitochondrial activity under UV stress. Similar responses have been reported in other insects under UV exposure [25,26]. For example, *Ostrinia furnacalis* exhibited enhanced energy metabolism, activation of antioxidant and detoxification systems, and suppression of non-essential processes related to development, neurology, and transcription upon exposure to UVA. This was accompanied by metabolic reprogramming and significant induction of detoxification genes such as *Cytochrome P450s*, *UGTs*, and *ALDHs* [27]. Likewise, in *Helicoverpa armigera*, short-term UVA exposure has been shown to activate the p38 MAPK signaling pathway, leading to the upregulation of antioxidant enzymes such as SOD, CAT, and GPX, along with a marked decrease in the GSH/GSSG ratio—indicative of oxidative stress [28,29,30].

The upregulation of mitochondrial pathways is often accompanied by the accumulation of reactive oxygen species (ROS), leading to oxidative stress. Our KEGG enrichment analysis revealed that UVA treatment significantly activated several critical pathways associated with ROS accumulation and oxidative stress responses. Mitochondria serve as the primary site of ROS production. The ROS generated within the mitochondria can not only induce oxidative stress but also trigger ROS-induced ROS release mechanisms that amplify oxidative signalling and influence cell fate decisions [31,32]. These findings suggest that UV radiation may enhance mitochondrial respiration and energy metabolism, consequently leading to elevated ROS production. Consistent with this, GO enrichment analysis in both UVA and UVC treatment groups indicated that upregulated genes were significantly associated with mitochondrial processes, such as aerobic respiration, and were localised to subcellular components, including the mitochondrial membrane and respiratory chain complexes.

In addition to enhancing mitochondrial activity and oxidative metabolism, UV irradiation significantly upregulated a suite of genes involved in detoxification and redox homeostasis, contributing to the insect’s molecular defence system against environmental stress. The cytochrome P450 enzyme system is widely distributed in the endoplasmic reticulum of animals and plants, playing a critical role in enabling insects to adapt to their chemical environment [33,34]. Cytochrome P450s serve as terminal oxidases in the microsomal electron transport chain and are key components responsible for xenobiotic metabolism [35]. Notably, P450 genes constitute one of the primary gene families promoting detoxification metabolism in insects [36]. A recent study by Du et al. demonstrated that *CYP6JM1*, a P450 gene, is highly expressed in the first- and second-instar nymphs and abdomens of *B. tabaci* [37]. This high expression contributes to resistance against insecticide thiamethoxam and plays a functional role in midgut detoxification. RNA interference-mediated knockdown of *CYP6JM1* expression resulted in increased sensitivity of *B. tabaci* to thiamethoxam, highlighting its pivotal role in regulating insecticide resistance. Another critical enzyme family involved in redox regulation is GSTs, which are known for their roles in metabolic resistance mechanisms and detoxification [38,39]. UV exposure has been shown to increase intracellular levels of reactive oxygen species in insects. In this context, GSTs help eliminate lipid peroxidation products and maintain cellular redox homeostasis, thereby enhancing insect tolerance to UV-induced oxidative stress [40].

The transcriptomic response to UVC treatment in this study shows a significantly weaker response, which may be attributed to several factors. First, UVC has limited penetration and primarily affects surface cells, thereby restricting the scope of the transcriptional response [41]. Second, UVC induces direct DNA damage, which can suppress the transcriptional machinery and lead to a weaker response [42]. This DNA damage may prioritise repair mechanisms over transcriptional activation, further diminishing the overall transcriptional response. Third, the high energy of UVC may rapidly deplete cellular energy reserves, leaving insufficient energy for comprehensive transcriptional processes. Lastly, the genes analysed in our study are mainly associated with oxidative stress, while UVC primarily induces DNA damage. In contrast, UVA primarily activates transcriptional responses through oxidative stress pathways, which may explain why the transcriptional response to UVC is weaker than that to UVA.

Therefore, the response pattern observed in our study, which enhanced mitochondrial metabolism and antioxidant capacity while suppressing non-essential developmental and transcriptional functions, may represent a conserved and adaptive strategy across insect taxa. This work not only provides new insights into the molecular mechanisms by which insects adapt to UV-induced stress but also offers a theoretical foundation for further research in insect stress physiology, biological control, and environmental adaptability.

In summary, this study employed a combination of RNA sequencing and quantitative real-time PCR to systematically investigate the differential gene expression patterns and potential biological functions in *S. alternatusi* Yang under shortwave (UVC) and longwave (UVA) ultraviolet irradiation. The findings revealed a typical stress-responsive transcriptional program characterised by the upregulation of mitochondrial energy metabolism and antioxidant defence networks, which contribute to cellular stability, alongside the downregulation of developmental and transcriptional regulatory functions to reallocate metabolic resources toward survival. This regulatory pattern suggests a strategic metabolic reprogramming in response to UV stress, which enhances defence capacity while suppressing energetically costly, non-essential biological processes. These insights not only enrich our understanding of the molecular mechanisms underlying UV stress responses in insects but also offer a theoretical foundation for the development of novel biological control strategies against *M. alternatus*. By considering the UV stress responses of natural enemies like *S. alternatusi*, such strategies could be optimised for controlling *M. alternatus*, especially in situations where UV exposure plays a key role. It can aid decision-making for integrated pest management by optimally selecting parasitoids based on specific environmental conditions.

## 4. Materials and Methods

### 4.1. Insect Source and Rearing Conditions

The *S. alternatusi* used in this study was provided by the Institute of Forest Ecology, Environment and Protection, Chinese Academy of Forestry. In the laboratory, the parasitoid was continuously reared using *T. gebleri* larvae as substitute hosts. All colonies were maintained under standardised conditions: a temperature of 25 ± 1 °C, a relative humidity of 70 ± 10%, and a photoperiod of 16L:8D.

### 4.2. Ultraviolet Radiation Treatments

Newly emerged adult *S. alternatusi* (within 24 h post-eclosion) were exposed to ultraviolet radiation at two wavelengths: UVA (365 nm) and UVC (253.7 nm), using 8 W UV lamps positioned 10 cm above the samples at an irradiance of 1600 W/m^2^. The exposure duration was 9 h for each treatment. Furthermore, the UV irradiation was conducted in a darkroom to ensure strict control of experimental conditions. The control group (CK) received no ultraviolet radiation. Following irradiation, 10 individuals per treatment were randomly selected and pooled, with four biological replicates per group [43]. All samples were immediately preserved in TRIzol reagent and stored at −80 °C for subsequent RNA extraction.

### 4.3. RNA Sequencing, Library Construction, Read Alignment and Assembly

Total RNA was extracted from *S. alternatusi* individuals subjected to different UV wavelengths using the TRIzol reagent. The quality of RNA extraction is evaluated using an agarose gel and a bioanalyser (Shanghai Jiapeng Technology Co., Ltd., Shanghai, China). The concentration of RNA is determined using Qubit 2.0, as mentioned in our earlier studies [44,45]. Strand-specific RNA-seq libraries were constructed, and paired-end sequencing was performed on a high-throughput platform. Base-calling was used to convert the raw image files into raw data, which were then subjected to rigorous quality control, including filtering of low-quality reads, removal of adapter contamination, and elimination of ambiguous bases. Only high-quality clean reads (Q30 ≥ 90%) were retained for downstream analyses.

Clean reads were aligned to the reference genome using appropriate aligners, generating BAM files for subsequent processing. Based on genome annotations, transcript assembly, and expression quantification, a comprehensive gene expression matrix was generated for all samples to facilitate differential expression analysis.

### 4.4. Differentially Expressed Genes (DEGs) Analysis

Differential gene expression analysis was conducted using the DESeq2 package (v1.44.0) [46]. Genes with an absolute value of |log_2_FoldChange| ≥ 1 and an adjusted *p*-value (*p*adj) ≤ 0.05 were considered significantly differentially expressed. Volcano plots were used to visualise overall expression changes in DEGs, and Venn diagrams were constructed to identify overlapping DEGs between treatment groups.

Functional annotation of DEGs was carried out using Gene Ontology (GO) enrichment and Kyoto Encyclopedia of Genes and Genomes (KEGG) pathway enrichment analysis [47]. Specifically, the GO enrichment analyses and KEGG pathway enrichment analyses were carried out using the R package clusterProfiler (v4.12.6). Protein sequences of DEGs were further submitted to a locally installed InterProScan (v5.67-100) for protein domain annotation, with the E-value threshold set at <1 × 10^−5^. Based on biological relevance and significance, four representative DEGs were selected for RT-qPCR validation.

### 4.5. RT-qPCR Validation

To validate the transcriptomic data, four genes that were significantly differentially expressed (Table 2) were selected for quantitative reverse transcription PCR (RT-qPCR). Gene-specific primers were designed using Primer3 (v.0.4.0; http://bioinfo.ut.ee/primer3/ (accessed on 11 August 2025)), and *Serine and arginine-rich splicing factor 7* (*SRSF7*) was used as the internal reference gene for normalisation [48].

To assess RNA integrity, a subset of total RNA was analysed by 1.5% agarose gel electrophoresis in 1× TAE buffer under 100 V for approximately 25 min. The clarity and integrity of 28S and 18S rRNA bands were used to confirm sample quality. RNA concentrations were measured using a spectrophotometer. First-strand cDNA synthesis was performed using the TB Green^®^ Premix Ex Taq™ II reverse transcription kit (Beijing Labgic Technology Co., Ltd., Beijing, China), following the manufacturer’s protocol: incubation at 42 °C for 2 min, 37 °C for 10 min, and termination at 85 °C for 5 s.

The RT-qPCR reaction (10 μL total volume) contained 1 μL primer mix (0.5 μL of each primer at 10 μM), 4 μL of 10-fold diluted cDNA, and 5 μL of TB Green Premix. Amplification was performed using the following cycling parameters: 95 °C for 30 s (initial denaturation), followed by 40 cycles of 95 °C for 5 s and 60 °C for 30 s. Melt-curve analysis was performed to confirm the specificity of the amplification products.

**Figure 5:**
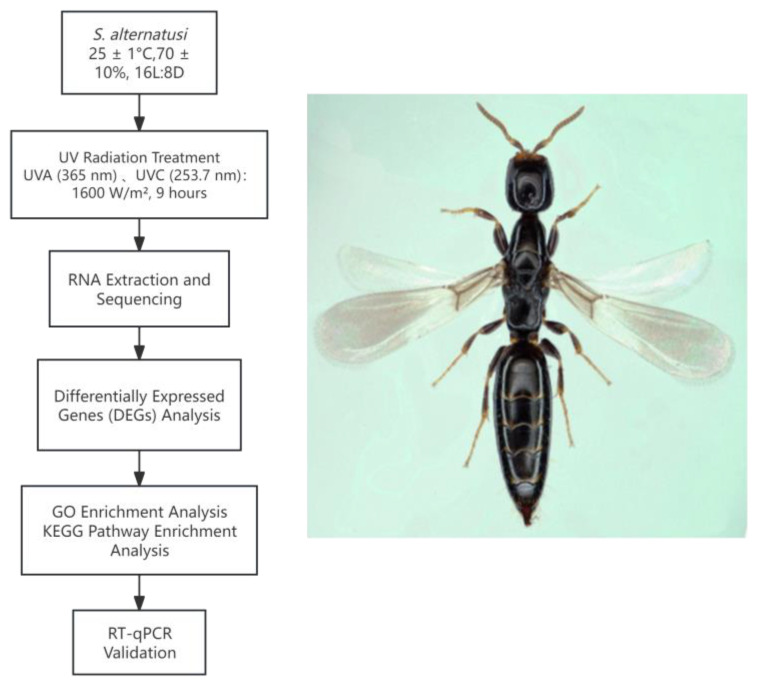
Experimental workflow and a representative image of *Sclerodermus alternatusi* Yang.

Each treatment group included four biological replicates, with technical triplicates for each sample. Cycle quantification (Ct) values were automatically calculated, and relative gene expression was analysed using the 2^−ΔΔCt^ method proposed by Pfaffl [49], with *SRSF7* used for normalisation. The statistical analysis of RT-qPCR data was performed using Microsoft Excel 2021, Microsoft Corporation, Redmond, WA, USA, while plotting was carried out using Origin 2021, OriginLab Corporation, Northampton, MA, USA. The one-way analysis of variance (ANOVA) followed by Scheffe’s post hoc test was performed using SPSS 17.0, IBM Corporation, Armonk, NY, USA. Experimental workflow and representative image of *S. alternatusi* is shown in Figure 5.

## Figures and Tables

**Table 1:** Summary of Quality Control and Mapping Statistics for RNA-Seq Data of *Sclerodermus alternatusi* Yang under UV Radiation Treatments.

Sample	Clean Reads	Q20 (%)	Q30 (%)	Clean Bases (Gbp)	GC Content (%)	Mapping Rate (%)
UVA_1	21,062,256	99.04	96.91	3.16	37	98.83
UVA_2	21,640,086	98.98	96.76	3.24	38	98.81
UVA_3	21,278,254	99.04	96.95	3.19	37	98.87
UVA_4	21,335,686	99.01	96.83	3.20	37	98.88
UVC_1	21,631,499	99.03	96.90	3.24	38	98.84
UVC_2	20,626,381	98.98	96.75	3.09	38	98.85
UVC_3	21,393,148	99.05	96.95	3.20	38	98.74
UVC_4	24,045,284	99.08	97.04	3.59	38	98.88
CK_1	19,689,673	99.03	96.87	2.95	39	98.86
CK_2	21,484,229	99.00	96.84	3.22	39	98.86
CK_3	21,232,268	98.97	96.75	3.18	38	98.75
CK_4	21,350,101	99.03	96.89	3.19	38	98.85

**Table 2:** Differentially Expressed Genes and Their Specific Primers.

Gene Name	Primer Sequences (5′−3′)	Length (bp)	Annealing Temperature (°C)
*Serine and arginine rich splicing factor 7*	FW: GGGTCGCTAGAAATCCTCCA	88	57.94
	RV: ACTCCATCCAAGCCACGAAC		
*Glutathione S-transferase 1*	FW: GAGATTGTGGAGAATGGAATGC	102	53.65
	RV: CGGATGAATAGGATGGTCTAGC		
*Alpha-ketoglutarate dehydrogenase component 4*	FW: TGTCTTGCCAGCAATAGATGAT	103	54.84
	RV: CGGTCCTCCACGGTTAATATAC		
*Cytochrome P450 304a1*	FW: GCTGTGCCAAGTAGTGTTACA	104	55.03
	RV: CTGCCTGTGCCAACAATAGAA		
*NADH dehydrogenase (ubiquinone) B22 subunit (ND-B22)*	FW: AATACGTGCTAGGTTCGATGAA	82	53.24
	RV: TTCTTCTTCTCCCGCTAACAAT		

## Data Availability

The original contributions presented in this study are included in the Supplementary Materials; further inquiries can be directed to the corresponding author. The RNA-seq raw reads were submitted to NCBI under Bioproject PRJCA050974.

## Supplementary Materials

The supporting information can be downloaded at: https://www.mdpi.com/article/10.3390/ijms27031163/s1.
